# Do Attitudes Towards Immigrants Matter? The Subjective Wellbeing of Immigrants in England and Wales and Their Exposure to Non-migrants

**DOI:** 10.1007/s10680-023-09686-z

**Published:** 2023-12-11

**Authors:** Michaela Šedovič

**Affiliations:** Department of Social Policy, London School of Economics and Political Science, Old Building, Office 1.19, Houghton Street, London, WC2A 2AE UK

**Keywords:** Attitudes towards immigrants, Subjective wellbeing, Integration of immigrants, Life satisfaction, England and Wales

## Abstract

The wellbeing of immigrants is affected by those around them and the context in which they live. Yet we still know relatively little about the impact that attitudes towards immigrants (ATI) have on immigrants’ life satisfaction, nor do we know the routes by which it manifests. By matching individual data from the UK Understanding Society study to area-level data on ATI for England and Wales from the 2018 European Values Study, I examine whether subnational ATI are associated with immigrants’ life satisfaction. If so, I aim to determine the geographical level at which it is prominent and identify the channels through which this association operates. By exploiting the different geographical scales at which ATI are aggregated, I show within-country variation in ATI. Controlling for contextual- and individual-level characteristics, I find that immigrants’ wellbeing is sensitive to exposure to the negative ATI of non-migrants at the regional level but not at the municipal level. Theoretically identified channels (local social cohesion and ethnic composition) are not drivers of this association, but it is moderated by (interethnic) friendships. Further, I show that ATI are a measure of environment rather than a function of intergroup contact or exposure and that the entire composition of the ATI in an area is more important than the most negative attitudes. I discuss the implications of these findings.

**Supplementary Information:**

The online version contains supplementary material available at 10.1007/s10680-023-09686-z.

## Introduction

Understanding the subjective wellbeing of immigrants is an important contemporary issue. Firstly, life satisfaction^1^ is a measure of an individual’s experience, which in this case concerns immigrants and their ability to live happily in the destination. Secondly, when we focus on immigrants as a group, their wellbeing and the conditions that improve or diminish it serve as indicators of a country’s success in creating effective integration policies and providing support for immigrant populations.

The effective integration of immigrants is a critical issue in Western Europe, due to the growing shares of settled immigrant populations (Hendriks & Bartram, 2019). Integration is typically examined in terms of immigrants’ success on objective measures, such as educational attainment, earnings, or mastery of the language (Bartram, 2011; Vervoort et al., 2012). Increasingly, it is argued that integration should (also) be assessed according to subjective criteria such as life satisfaction (Hendriks & Bartram, 2019; Jenkins, 2020). Such measures may better reflect an immigrant’s own evaluation of the success of their migration project (Baykara-Krumme & Platt, 2018). In addition, life satisfaction does not necessarily correlate with objective criteria. For example, Bartram (2011) finds only a weak association between immigrants’ total income and their self-assessed life satisfaction. This raises the question of whether objective (particularly economic) measures are sufficient for assessing the success of migration projects. To identify what contributes to immigrants’ own sense of success in the destination, we need to understand the additional factors that influence their life satisfaction.

I analyse the relationship between immigrants’ expressed life satisfaction and local non-migrants’ attitudes towards immigrants (ATI). One critical influence on immigrants’ wellbeing is their lived environment. Integration is a two-way process (Klarenbeek, 2021), and a welcoming or hostile environment can affect an individual’s ability to integrate. Hostile environments are associated with social isolation (Maggio, 2021) and immigrants feeling like outsiders (Berry, 1997). Perceived and/or experienced discrimination lead to lower wellbeing and diminished mental health (Nandi et al., 2020), and their consequences need to be researched further (Esses, 2021). Without an environment that promotes (positive) exposure to non-migrants, immigrants cannot acculturate to their new society (Vervoort et al., 2010). Therefore, it is crucial to examine immigrants’ wellbeing and how non-migrants can affect it.

The research on the impact that the exposure to non-migrants has on immigrants’ wellbeing is neglected in current literature. Most research on immigrants’ wellbeing which considers non-migrants as a factor, attributes the variation to the immigrants’ individual perceptions of discrimination or non-migrants’ behaviour. These tend to be associated with immigrants’ lower wellbeing (Kirmanoğlu & Başlevent, 2013; Obućina, 2012; Safi, 2010; Verkuyten, 2008; Vohra & Adair, 2000). However, measures of perceived discrimination capture only negative interactions, and since they are based on subjective perceptions, they might be endogenous to other subjective measures such as wellbeing. Another approach uses proxy measures of contact, which assume that contact occurs when immigrants and non-migrants are in proximity and affect each other (Knies et al., 2016; Sapeha, 2015). These studies employ measures such as ethnic composition or foreign population levels. Proxies offer a greater potential to capture the extent of contact and exposure to non-migrants, but they generally lack information about whether the interaction is positive or negative. The research that considers non-migrants’ attitudes tends to take the perspective of methodological nationalism and treats a country’s population as homogenous (Heizmann & Böhnke, 2018; Kogan et al., 2018).

Although ATI are not a measure of contact, I consider local ATI as a measure of exposure to outgroup and as a complementary tool to existing measures, namely proxies and experienced discrimination. This allows me to better explore the meso-level and contextual level. Contemporary research in the field of contact theory highlights a lack of investigation into the meso-effect, specifically the effect of contact within the lived context. Various authors propose that a person living in a context with a higher mean level of positive contact can experience positive outcomes from these intergroup interactions. These outcomes may extend beyond their own contact and remain independent of any knowledge about others having experienced positive intergroup interactions (Hewstone, 2015).

Local ATI overcome the limitations of previously used measures in capturing the impact of non-migrants on immigrants’ wellbeing in four ways. Firstly, ATI capture both the positive and negative spectrum of attitudes. Secondly, ATI are not endogenous to wellbeing, as an individual immigrant’s wellbeing is unlikely to affect ATI in a given area. Thirdly, they potentially capture non-migrants’ responses to immigrants in ways that go beyond specific types of behaviour, such as voting. Except for certain isolated groups, immigrants interact with the population of their destination country in various situations on a daily basis. It would be impossible to capture and measure them all. Thus, ATI provide a more general description of the lived environment of immigrants. Lastly, local ATI allow us to observe the within-country differences in non-migrant attitudes.

Immigrants experience attitudes through contact and exposure to non-migrants, namely by having them as friends, neighbours, and colleagues, or simply by residing in the same spaces, neighbourhoods, or regions. All these channels of exposure can therefore be associated with better or worse wellbeing. However, the literature is inconclusive on the direction of associations, with the results differing across studies. This could be explained by the diverse character of contact and varying degrees of exposure that are studied. The character of contact and exposure may be either positive or negative. I capture the character of contact and exposure using ATI on local and regional levels and test some of these channels.

Using a nationally representative study of the UK with large samples of immigrant groups, I employ regression models to estimate the association between local and regional aggregated ATI and self-reported life satisfaction. Multiple levels of ATI allow me to identify which theoretical channels of exposure influence the association with life satisfaction and, thus, which of these are its potential drivers. Examining multiple levels also reveals subnational differences in the relationships of immigrants with their environment.

My descriptive results reveal previously unaccounted for associations between ATI and subjective wellbeing at the regional level. With the exception of interethnic friendship, for which results suggest some moderating effect, these associations are not influenced by the other potential channels I explore, namely social cohesion and ethnic composition. I discuss the implications of my findings.

## Background

Although certain determinants of life satisfaction, like employment, are the same for immigrants and non-migrants (Dolan et al., 2008; Kogan et al., 2018; Luttmer, 2005), other factors are unique to the particular experiences of immigrants. For example, identifying with the destination country, integration level, opportunities to integrate, and discrimination (Crul & Schneider, 2010; Hendriks & Bartram, 2019; Safi, 2010; Vohra & Adair, 2000). Many of these factors are linked to immigrants’ social relations and environment in the destination. This includes the networks and (in)groups they belong to (Arpino & de Valk, 2018; Sapeha, 2015), their contacts (Sapeha, 2015), and their exposure to non-migrants in spaces that both groups occupy simultaneously (Hellgren, 2018; Kirmanoğlu & Başlevent, 2013; Knies et al., 2016; Wiedner et al., 2022).

The effect of intergroup exposure on individual wellbeing is influenced by two important determinants. First is the character of the exposure, determining if it is positive or negative. Second is the extent of exposures, determining the level and frequency of exposure, which may depend on several aspects of destination such as migrant-group concentration and also one’s social contacts.

### Character of Exposure

According to intergroup contact theory (Allport, 1958; Pettigrew & Tropp, 2011) and empirical research on immigrant and non-migrant samples (Laurence et al., 2018), the character of exposure can be positive, negative, or ambiguous. Therefore, this exposure could affect certain aspects of immigrants’ lives positively, negatively, or to varying magnitudes. Thus, distinguishing the character of exposure is essential in identifying the direction of the relationship effect between groups (Allport, 1958). The same also holds true for research on immigrants’ wellbeing.

Research shows that negative attitudes and behaviours towards immigrants are associated with their mental and physical wellbeing. For example, Kogan et al. (2018) conducted a comparative study of 18 European countries in which they argue that more racist ATI threaten immigrants’ wellbeing. Nandi et al. (2020) found that the experience of harassment had an adverse effect on the mental health of immigrants in the UK and increased their anxiety. Perceived discrimination globally serves as an explanatory factor for immigrants’ lower life satisfaction (Safi, 2010; Vohra & Adair, 2000). For example, Schilling and Stillman (2021) show that exposure to far-right mobilisation negatively impacts asylum seekers’ integration. Furthermore, Wiedner et al. (2022) demonstrate its impact on the wellbeing of immigrants in Germany. This is especially true for skilled immigrants (Knabe et al., 2013).

On the other hand, Kogan et al. (2018) associate more positive national ATI with higher life satisfaction among immigrants. Similarly, qualitative studies argue that living in more inclusive areas alleviates immigrants’ feelings of disintegration and detachment (Hellgren, 2018), two factors that are closely linked to life satisfaction (Amit, 2010).

One common feature of research based on the character of contact is the use of subjective measures to indicate perceived discrimination, such as feeling discriminated against or self-assessing oneself as belonging to a discriminated group. Another is the use of non-migrants’ specific behaviours, like voting patterns or performed discrimination. There are two main reasons why measures capturing immigrants’ perceptions might inadequately describe or introduce bias when assessing information about the lived environment of immigrants. First, there are issues with the measurements themselves, as they capture only negative perceptions and might be endogenous if related to other subjective measures. Second, there are issues with the data collection, as survey questions might be too specific and thus collect only information about particular encounters. Immigrants might not feel comfortable answering these questions. Some may not experience or perceive discrimination themselves but may still be affected by the experiences of fellow immigrants. For instance, Hopkins et al. (2016) show very little geographical variation in perceived discrimination in the USA, despite differences in the behaviour and anti-immigrant attitudes of residents. They suggest that perceived discrimination might not be perceived in the immediate environment while also finding that its triggers are unclear and that it might be decoupled from non-migrants’ behaviour. Measures that capture non-migrants’ behaviour may be better than perceptions at partially describing the environment. However, immigrants’ perceptions may be influenced by specific non-migrants’ behaviours other than voting preferences or support for a political party. Examples of such behaviour could be having a Brexit bumper sticker or asking an individual with an accent where they are from. It is difficult to capture the general behaviour of non-migrants as a sum of all their actions by using narrowly specified measures. What is more, they do not indicate how such behaviours are actually observed or experienced by immigrants.

To conclude, we lack a comprehensive understanding of the association between subjective wellbeing and the character of contact or exposure, as they are measured through own perceptions of immigrants or other inadequate measures. Moreover, these studies are often conducted on the national level (Kogan et al., 2018; Safi, 2010), which might obscure the association, if lower level exposure is relevant for immigrants’ wellbeing.

### Extent of Exposure

Immigrants can experience exposure to non-migrants in various ways, such as through personal contact, neighbourhood interactions, in the workplace, commuting, or formally in institutions. The workplace and local residential area are the two primary settings where people spend their lives (Laurence et al., 2018). Therefore, many of these exposures to others occur there (Laurence, 2013). However, the research is inconclusive regarding whether higher or lower immigrant/own-group concentration in local areas positively or negatively impacts immigrants’ wellbeing. A research study on 15 western and southern European countries showed a strong negative correlation between life satisfaction and local ethnic diversity for both immigrants and non-migrants (Davies et al., 2011). The results suggest that increased ethnic diversity is connected to ethnic and religious tensions and that UK residents are more sensitive than other countries to any changes in their local environment.

However, Knies et al. (2016) do not find this pattern. Using UKHLS, they find variation in the association between life satisfaction and own-group ethnic concentration. Some groups (Pakistanis) report lower life satisfaction, while others (Black Africans and second-generation Indians) report higher levels. A recent German study used a novel dataset with measures of ethnoreligious density based on places of worship and ethnic businesses to find associations between higher wellbeing and greater ethnoreligious density, especially for non-European immigrants (Wiedner et al., 2022). In contrast, the regional concentration of immigrants is negatively associated with the life satisfaction of immigrants in Canada (Sapeha, 2015). The same study shows higher levels of satisfaction among immigrants with more interethnic friendships.

The generally accepted explanation for differences in results is that some groups benefit from own-group concentration in the form of protection (Cobb et al., 2019), whereas others benefit from exposure to the destination country’s culture and non-migrants, as it speeds up their integration. Furthermore, this relationship may vary over time. For example, living primarily within the immigrant’s own-group might initially provide benefits such as developing skills and building networks, but this could later prove to be an obstacle to improving their economic advancement (Musterd et al., 2008), language proficiency (Vervoort et al., 2012), or links with non-migrants (Vervoort et al., 2010).

However, the association might also be explained by whether immigrants are exposed to hostile or welcoming environments, as suggested by research on the character of contact. We cannot confirm this assumption because the extent of exposure and its character are studied separately. Firstly, proxies of exposure like neighbourhood diversity measure the extent of exposure but do not capture its character. Secondly, the research on the character of exposure produces results that may not be generalisable to all immigrant populations, but rather to those who self-assess as experiencing discrimination or being members of such marginalised groups. The combination of these two factors produces a knowledge gap. Therefore, I employ non-migrants’ attitudes towards immigrant as a measure of exposure and to capture its character. I further test, whether the measures of the extent of the contact interact with the association and channel the ATI of non-migrants (see subsection *Research framework*).

### Attitudes Towards Immigrants

According to Reitz (2002), attitudes towards immigrants provide a set of pre-existing boundaries within which integration takes place in the destination. Average ATI measures the mean level of positive or negative interactions in an area, which predict the social norms of valuing or not valuing diversity (Hewstone, 2015). Thus, I assume the measure of ATI encompasses behaviours towards immigrants to some extent. This includes behaviour such as voting, but also more subtle expressions of pro/anti-migrant behaviour that would be harder to capture in other ways. The non-migrants’ ATI might also be seen as a proxy for legal regulations and policies, which they informally create by influencing policy makers (Reitz, 2002). However, although ATI encompasses other behaviours, it also has the advantage of being an important measure on its own. Immigrants might be affected by ATI, even if they are not acted upon, simply by knowing these attitudes. For instance, EU immigrants feel more fearful in the UK after the Brexit referendum, despite no evidence of any increases in intergroup violence (Nandi & Luthra, 2021). The results of the referendum informed immigrants of these particular attitudes. However, the election results are not the only way for immigrants to observe the ATI of non-migrants, considering they are in daily contact.

While ATI are a well-researched phenomenon from the non-migrants’ perspective (Davidov et al., 2019; Meuleman et al., 2009), they are under-researched from the immigrants’ perspective (Becker, 2019; Ramos et al., 2019). Non-migrants’ ATI are even more rarely employed as determinants in research analysing immigrants’ life outcomes. Two cross-national studies explore the impact of ATI on immigrants (using the European Social Survey). In the first, Heizmann and Böhnke (2018) use ATI to measure symbolic boundaries between the natives and immigrants. In the second, Kogan et al. (2018) focus on welcoming environments, which they measure through both aggregated ATI and legal migrant integration regulations and policies (MIPEX).

While these two studies confirm an association between wellbeing and ATI, both are international comparative studies and their unit of analysis is a nation-state, meaning that ATI is aggregated at a broad level. Kogan et al. (2018) test two determinants of wellbeing: 1) ATI and 2) integration policies. The legal regulations should serve as a better measure at the national level, as they do not vary across a country. Nevertheless, the authors refute the hypothesis that regulations are linked to wellbeing and find wellbeing has an association only with ATI, which exhibit notable cross-country variability.

Considering that these research studies do not account for within-country variability, their results point towards the necessity of a more granular approach to analysing the association with ATI, as we do not know whether within-country variation in attitudes is relevant in determining immigrants’ life satisfaction. Nor do we understand whether differences in life satisfaction align with the channels through which immigrants might encounter attitudes, as well as the factors that might mediate these associations.

### Research Framework

Building up on the research gaps identified in the studies of immigrants’ wellbeing and its association with the character and the extent of exposure, I study the subnational association of immigrants’ wellbeing and regional and local attitudes towards immigrants. I aim to study how character of exposure impact immigrants, when the character is captured using measures independent of immigrants’ perceptions and show whether there is within-country variation in the association.

My study is set in the UK. It is an interesting case study considering the recent importance of the immigration in the national politics and the role it played during Brexit. The topic of immigration has been since often discussed among general public and in media and thus influencing non-migrants and immigrants. Moreover, the UK is a research setting which, according to Platt and Nandi (2020), presents a considerably complex portrayal of immigrants’ experiences. The UK exhibits substantial demographic and socioeconomic diversity within and between immigrant groups, and its long immigration history enables comparing the wellbeing of diverse immigrant groups and cohorts. Moreover, a substantial and growing body of literature is centred in the UK, encompassing research that explores topics similar to the subject of this paper, which allows me to situate my findings within the broader context of research on immigrants.

In my research design I consider different aspects of non-migrant behaviour towards immigrants. Of these, the most important concern where and how specific behaviours may manifest and be experienced by immigrants. Therefore, I aggregate the ATI at two levels: (1) local (NUTS3—comparable to Local Authority Districts (LAD)); and (2) regional (NUTS1/Government Office Region (GOR)). When aggregating attitudes, I presume they drive behaviour (Schuman et al., 1985), specifically behaviour towards immigrants (Malloy et al., 2021).

I choose to employ *the local level* for two reasons. First, it is reasonable to assume that is where immigrants spend the majority of their everyday life and thus experience most of their daily interactions, whether they be with locals or immigrants. Second, while the governance of immigration operates primarily at the (inter)national level, the governance of integration is progressively shifting towards local levels (Glick-Schiller & Çağlar, 2009; Hackett, 2015). This recent “local turn” (Zapata-Barrero et al., 2017) in governance means that immigrants are increasingly influenced by the local environment and governments, which are primarily composed of and elected by non-migrants. Thus, research on the relationship between immigrants and their lived environment must also focus on this level. The focus on subnational levels also overcomes the issues of methodological nationalism and shows diversity within countries instead of treating them as homogenous units (Glick-Schiller & Çağlar, 2009). LAD is a policymaking level in the UK, which means that residing in a particular district can specifically affect one’s life.

Two issues emerge when using LAD aggregation. Methodologically, the UKHLS contains only a small sample size of immigrants, which may lead to an increased margin of error and a lack of statistical power. I attempt to adjust for this by excluding units with excessively small samples. As this prevents me from analysing all LAD units, my analysis covers only a part of England and Wales, specifically urban areas. This in turn gives rise to the second issue, which concerns the intergroup relations in these areas. Research shows residents in urban areas might be disengaged from others, especially strangers (Zeeb & Joffe, 2021). This might show up in analyses due to immigrants’ and non-migrants’ possibly being ignorant of each other. Conversely, there may be a risk of person-positivity bias, in which individual’s negative attitudes towards an abstract outgroup do not necessarily translate into hostility towards members of that group (Iyengar et al., 2013; Sears, 1983). Person-positivity bias would mean disassociation between (negative) ATI and (hostile) behaviour and, thus, I would observe no association. Higher population densities and concentrations of immigrants in local urban areas might create the conditions that generate this bias. Therefore, my analysis also employs the GORs. Although *regional aggregated data* is not as good as LADs for measuring the immediate environment of an individual, regions are nevertheless distinct enough to capture the specificities of the environment in which individuals live. For instance, Devon is more comparable to Cornwall, which is in the same GOR, rather than to Essex or Northumberland, which are in other regions.

Existing theoretical and empirical research also supports the use of multiple levels of analysis. There is no agreement on the most appropriate spatial level for measuring interethnic interactions (Petrović et al., 2018) as exposure to others varies across different locations and at various scales (Manley et al., 2006), depending on the characteristics of particular areas. This implies that individuals may experience different environments when moving among regions. My research design allows me to capture potential inter- and intra-regional diversity while providing a more comprehensive understanding of the environment in which individuals live.

Many studies discuss the effect of neighbourhoods on immigrants (Knies et al., 2016; Wiedner et al., 2022). I decided against engaging neighbourhoods and the neighbourhood effect theory, due to the possibility that using such small units could cause endogeneity in my explanatory variable. Contact theory shows that individual attitudes are affected by interpersonal contact or the lack thereof. The life satisfaction of immigrants living in these small units could affect the ATI of non-migrants at the neighbourhood level, potentially leading to variations in ATI and introducing reverse causality. Choosing higher granularity allows me to assume that the aggregated ATI are not directly influenced by the life satisfaction of immigrants in those areas.

As I expect ATI to be related to subjective wellbeing, I investigate the channels which expose immigrants to non-migrants’ ATI. I test two widely employed determinants of subjective wellbeing, which characterise immigrants local lived environment—ethnic concentration and social cohesion and their role in the association between life satisfaction and ATI (Davies et al., 2011; Knies et al., 2016; Laurence & Bentley, 2016). Additionally, I investigate the role of intergroup friendships (Sapeha, 2015). While these might not be linked to the local environment, they serve as an indication of an individual’s socialisation outside of their own-group and thus of intergroup contact, which might influence the association of non-migrants’ ATI. Positive intergroup contact is a known determinant linked with understanding between groups (Pettigrew & Tropp, 2011). Having such friendships could be a predictor not only of the ability to empathise with one another’s circumstances, but also of a decrease in concerns about non-migrants’ ATI.

At a more granular local level, I test two area-specific determinants as channels of exposure: ethnic composition and social cohesion. I assume that the variation in their impact on wellbeing, as described in the existing research (Davies et al., 2011; Knies et al., 2016; Sapeha, 2015), is linked to differences in local and regional ATI. My hypothesis is that immigrants exposed to greater shares of white British citizens are also exposed to more negative ATI, thereby resulting in lower-reported life satisfaction. Cross-sectional studies argue that a diverse local environment (the extent of exposure to others) leads to negative outcomes in the community (Davies et al., 2011). Laurence and Bentley (2016) present a longitudinal analysis suggesting that preferences for or against outgroup neighbours (referring to the quality of the intergroup relations) may be the underlying reason for the varying impact of diversity on social cohesion. I hypothesise there is a potential for the same association: individuals living in areas with higher social cohesion are exposed to more positive ATI and report higher life satisfaction. Since local ATI map areas closest to an individual’s home, where I expect them to spend the majority of their time, I expect the relationship between ATI and wellbeing to be stronger there.

At the less granular level, I examine the share of interethnic friendships as a channel of influence. Previous research acknowledges their moderating effect on the association between environment and wellbeing (Laurence et al., 2018; Sapeha, 2015). I hypothesise a weaker association between ATI and wellbeing for individuals with interethnic friendships.

The pool of identified wellbeing determinants is naturally extensive and goes beyond the scope of this work. While I acknowledge them, my aim is not to offer a comprehensive analysis of all those determinants but rather enhance our understanding of the extent to which the environment shapes immigrants’ life satisfaction and the channels through which this influence occurs. I focus on the potential of an under-researched existing measure (ATI) and control for determinants, which might influence the association of interest.

## Data and Methods

### Data and Sample

I use Understanding Society—the UK Household Longitudinal Study (UKHLS) wave 9 (University of Essex, 2020). This dataset is matched at the area level in order to aggregate measures derived from the European Value Survey 2018 (EVS, 2021).^2^

The UKHLS is the nationally representative longitudinal household panel that provides data from all adult members (aged 16 and above) residing in approximately 40,000 households, encompassing around 100,000 individuals. Each adult member of a household is asked core questions in a face-to-face interview and through a self-completion online survey on an annual basis, supplemented by rotating modules. It is not only a representative study but also includes an Ethnic Minority Boost sample (since 2009) and an Immigrant and Ethnic Minority Boost sample (since 2014) to ensure adequate subsample sizes for analysing minority and immigrant groups.

My main analytical sample and all but explanatory variables come from the UKHLS data collected during the period 2017–2019. As I aim to analyse immigrants and the local areas where they live, I apply four criteria to restrict my sample: (1) adults (16+ years old) who were born outside the UK, with at least one parent born outside the UK, and who migrated to the UK at some point in their lives; (2) individuals who answered the question about their life satisfaction; (3) individuals from the NUTS3 units included in the European Value Survey, which provides ATI information; and (4) only those in the NUTS3 units with a sufficient number of observations (at least 30 per unit) to conduct the analysis at the local level (*N* = 2096). All other respondents are excluded from the sample. This resulted in streamlining my sample to mostly urban areas. The size of NUTS3 units ranges between 150,000 and 800,000 people. The missingness rates for individual variables range from 0.05 to 3.84%, with the exception of the education variable, which reaches 12.83%.^3^ For all variables except education, I use listwise deletion. The education variable is categorical, and I recode missing cases into a separate category to retain the sample size (Appendix 1).

The analytical sample is combined with the European Value Survey, which is an international cross-sectional survey. The EVS aims to provide representative data of the resident population aged 18 years and older, with the targeted national sample ranging between 1000 and 1500 individuals. The survey uses a probabilistic sampling method to gather representative data via face-to-face interviews, with mixed-mode methods included as an experimental component.

The UKHLS wave 9 data are suitable for my analysis because it is one of the three waves that include the neighbourhood module, which I employ to investigate channels of exposure. Additionally, the timing aligns with the European Value Survey 2018 data, which provides my explanatory variable. The EVS data offer the most recent available source of information on individuals’ ATI which also captures residency information at a geographical level smaller than the Government Office Region (Appendix 2).

### Measures

#### Dependent Variable

I use the self-reported life satisfaction to measure immigrants’ subjective wellbeing. This measure is based on a 7-point scale in answer to the question: *Please choose the number which you feel best describes how dissatisfied or satisfied you are with the following aspects of your current situation: Your life overall.* The scale ranges from *completely dissatisfied* (1) to *completely satisfied* (7). This measure captures individuals’ cognitive assessment of their life as a whole (Veenhoven, 2000) and is recommended for the study of outcomes related to immigration (Hendriks & Bartram, 2019). I decided against using other measures such as happiness as it is considered as a simpler measure of day-to-day positive emotion in contrast to life satisfaction measure (Veenhoven, 2000). Since my focus is on overall satisfaction (Veenhoven, 2012), I avoid using an index of life satisfaction dimensions, such as job satisfaction.

#### Independent Variable

The aggregated ATI at the regional and local levels are derived from the EVS. Local geographical areas NUTS3 mostly correspond to LADs, for instance, the London Borough of Croydon; however, some combine a number of LADs, for instance, the NUTS3 unit *Haringey and Islington* combines the London Boroughs of Haringey and Islington. NUTS1 regions are the same as GORs, (e.g., East of England). This aggregation yields the ATI values of 28 NUTS3 areas and 10 NUTS1 regions.

The EVS contains five items that measure ATI. One question asks for responses measured on a 5-point scale. In four statement pairs, respondents position themselves closer to the one they agree with more (Table 1).

**Table 1:** Variables measuring ATI in the European Value Survey questionnaire

	Variable	Scale	Scale orientation	Included in explanatory indicator
1	Now we would like to know your opinion about the people from other countries who come to live in Britain—the immigrants. How would you evaluate the impact of these people on the development of Britain?	5-point scale	1-very bad 5-very good	In robustness checks only

	Matrix of statements	Scale	Orientation of the scale	
2	Immigrants take jobs away from the British—immigrants do not take jobs away from the British	10-point scale	1-completely agree with negative statement 10-completely agree with positive statement	Yes
3	Immigrants make crime problems worse—immigrants do not make crime problems worse			Yes
4	Immigrants are a strain on a country’s welfare system—immigrants are not a strain on a country’s welfare system			Yes
5	It is better if immigrants maintain their distinct customs and traditions—it is better if immigrants do not maintain their distinct customs and traditions			No

I investigated these measures using correlation and factor analyses. Based on the results (Appendix 3), I combine variables 2, 3, and 4 into a continuous indicator that measures attitudes on a 10-point scale, ranging from 1 (the most negative) to 10 (the most positive) (Cronbach’s alpha = 0.94). I excluded statement 5 due to its ambiguity and lack of correlation with the other variables. Measure 1, which is scaled differently, is not used in the main indicator. Nevertheless, I use a rescaled 5-point index that includes variables 1 to 4 as a robustness check.

The local ATI scores range between 3.3 and 10 points, while the regional ATI scores range between 4.6 and 6.2 (both on a 10-point scale). Non-migrants in the Greater London region exhibit the most positive regional ATI scores, while the most negative are found in the north of England. However, at the NUTS3 level, variation is high within the GOR areas. The higher variation at the more granular level aligns with my theoretical expectations of a stronger association in those areas.

My main analysis uses averaging as the method of data aggregation (cf. Heizmann & Böhnke, 2018; Kogan et al., 2018). However, I also run separate models using other methods of aggregation to check for the robustness of results and investigate if the potential association is driven by the most negative ATI (see Robustness checks). Specifically, I aggregate ATI using the mode, median, and share of negative attitudes in population (share of respondents indicating the most negative attitudes with 1 and 2 on a 10-point scale, where 10 is the most positive).

#### Control Variables

In order to isolate the association between ATI and wellbeing from other effects, I employ control variables. Employing individual- and regional-level controls allows to explain the variations in the strength of association between and within immigrant groups. It is clear from both international and UK research that ethnically visible immigrants have lower life satisfaction (Amit, 2010; Safi, 2010; Wiedner et al., 2022). Potentially, it is because of different treatment of non-migrants lower life satisfaction in their countries of origin. Thus, I expect that the variation in the association that depends on the area of origin is due not only to different exposure levels but also to the character of contact, as non-migrants might have different attitudes towards various immigrant groups. I also control for the origin of immigrants, as self-selection in immigrant settlement patterns and the composition of immigrant groups can influence the variation in ATI within specific areas, especially if they are the dominant minority.^4^ I focus on factors that could be linked to life satisfaction, and the non-migrant population’s perception of individuals (e.g., cultural background/origin) and/or can expose them to non-migrants (e.g., social activities, being employed). Finally, I control for individual and contextual factors such as the area's sociodemographic and economic characteristics (Knies et al., 2016; Musterd et al., 2008; Paparusso, 2018), as they might influence individual wellbeing and ATI, despite their limited support for the economic threat theory (Hendriks et al., 2022).

Thus, I include the following individual immigrant characteristics in my analysis: employment status (binary), social interactions (binary), region of origin (5 categories), length of stay in the destination (3 categories), sex (binary), age (continuous) and its quadratic term, and highest level of education attained (seven categories). I also control for regional unemployment rate. Additionally, as channels of exposure, I incorporate neighbourhood cohesion (measured using Buckner’s Neighbourhood Cohesion Instrument—short), local area ethnic composition measured as the proportion of White British residents, and having friends from another ethnicity (three categories) (Table 2).

**Table 2:** Descriptive statistics of all explanatory and control variables

		Suitable NUTS3 units	
		*N*	%
Life satisfaction	Least satisfied	55	2.62
	2	111	5.30
	3	170	8.11
	4	295	14.07
	5	432	20.61
	6	781	37.26
	Most satisfied	252	12.02
Sex	Male	913	43.56
	Female	1183	56.44
Age	mean/SD	48.31	15.44
Place of birth	Europe, Australia, North America	245	11.69
	India, Pakistan, Bangladesh	873	41.65
	Africa	237	11.31
	South America	93	4.44
	Other	648	30.92
Length of stay in the destination	0–5 years	94	4.48
	6–19 years	834	39.79
	20+ years	1168	55.73
Education	Lower secondary and lower	237	11.30
	Upper secondary	241	11.50
	Higher education	226	10.78
	University	650	31.01
	Other	473	22.57
	Missing	269	12.83
Job	Unemployed and out of labour	874	41.7
	Employed	1222	58.3
Social meetings	No	358	17.08
	Yes	1738	82.92
Interethnic friendships	No friends	75	3.58
	More than half same	1330	67.03
	Half or less the same	691	32.96
Social cohesion (Buckner)	Mean/SD	3.54	0.77
Ethnic composition (Share of British White residents)	Mean/SD	56.17	20.68
GOR unemployment rate	Mean/SD	4.81	0.74
Total		2096	

Data on the unemployment rate and ethnic composition rate are sourced from ONS (2018)

### Empirical Strategy

I estimated two sets of linear regression models. In both of them, life satisfaction (measured on a 7-point scale) was regressed on aggregated attitudes (10-point scale) while controlling for individual and regional characteristics. I first estimated ordered logistic regression models (Appendix 4), treating the response variable as an ordered categorical variable (for the discussion on wellbeing measures see Jenkins, 2020). I then compared these results with the results estimated in linear regressions. Since the results were comparable and linear regressions are easier to interpret (especially when using the interaction term), I present the results from the linear regressions. After assessing the limited number of individual observations, the observed NUTS3 regions, and the discussions on multilevel modelling (e.g., see Bryan & Jenkins, 2016), I concluded that the sample size prevents me from using multilevel modelling and thus opted for linear models.

I analysed the data first in models where the main explanatory variable was aggregated at the NUTS3 level, and then in a model where attitudes were aggregated at the GOR level. The models using attitudes aggregated at the NUTS3 level included the GOR as a fixed effect to control for variations in regional characteristics. Given the complex survey design of the UKHLS, I adjusted my estimates to account for stratification, clustering, and non-response weights using the “svyset” Stata command. For wave 9, I used the UKHLS weights, which were specifically designed for cross-sectional research of a single wave. In the models with the explanatory variable at the NUTS3 level, I cluster standard errors at that level (Moulton, 1990).

#### Variation in the Association

As discussed in the Background section, I test three channels of exposure. As they are linked to respondents’ residential areas, I test the interaction terms of *Neighbourhood cohesion* and *Ethnic concentration* with NUTS3 level attitudes. Then, I test the moderating effect of *Interethnic friendship* at a higher geographical level, as this channel is not specific to a geographical area.

## Results

### Descriptive Results

**Fig. 1:**
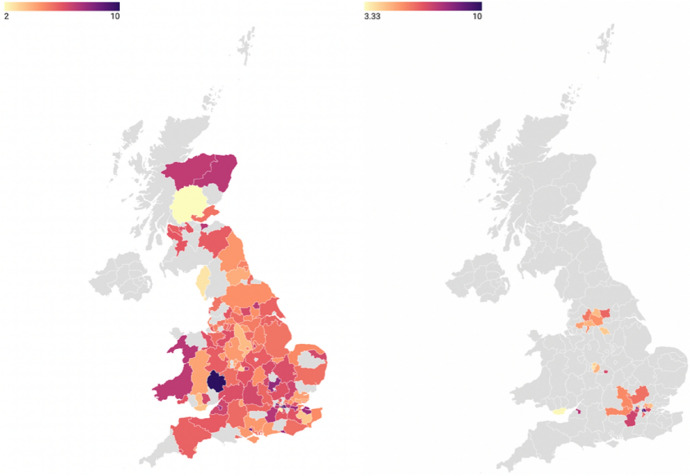
Aggregated ATI at the NUTS3 level. The grey areas on the left map represent missing data from the EVS. The grey areas on the right map additionally indicate regions with fewer than 30 observations per unit. *Note*: The left map illustrates the variation in ATI across the country. The right map shows variation in ATI for the examined sample. Darker areas indicate more positive attitudes towards immigrants

The sample primarily consists of highly populated and urbanised areas, namely London, Bristol, Manchester, Leeds, Sheffield, Birmingham, and Cardiff (Fig. 1—right). Although these areas may not be representative of the entire population of England and Wales, they do represent areas where most immigrants live (Knies et al., 2016). Therefore, I generalise my findings to the immigrant population residing in these areas.

### OLS Estimates

Table 3 presents estimates from models using local attitudes, where higher values indicate more positive attitudes. Model 1 represents the unadjusted association, and Model 2 is the full model. Model 3 controls for the GOR, and Model 4 incorporates all three channels of exposure simultaneously. Across all four models, there is no significant association between local ATI and life satisfaction. These results suggest that local attitudes do not play a role in determining immigrants’ wellbeing. They potentially align with theories proposing disengagement between individuals in urban areas (Zeeb & Joffe, 2021) and the person-positivity bias.

**Table 3:** Linear regression model estimates of immigrants’ life satisfaction on local ATI

	Model 1	Model 2	Model 3	Model 4
	Unadjusted	Full model	Full model with GOR	Channels
Local ATI	0.034	0.012	0.015	0.009
	(0.026)	(0.027)	(0.022)	(0.027)
Share of White British residents				0.005*
				(0.003)
Social cohesion				0.331***
				(0.043)
Half or less friends same				− 0.070
*(r.c. More than half friends same)*				(0.070)
No friends				− 0.440
				(0.182)**
Individual controls		Yes	Yes	Yes
GOR region			Yes	Yes
*R*^2^	0.00	0.04	0.05	0.08
*N*	2096	2096	2096	2096

***p < 0.01; **p < 0.05; *p < 0.1. All analyses are adjusted for sample design and non-response. Controls not shown in the table: sex, age, age squared, education, employment, region of origin, length of stay in the destination, socialisation, and dummy for GOR. Full models in Appendix 5

Regarding the potential channels, there is a small but significant positive association between higher wellbeing and the proportion of white British residents in the local area. Their concentration as an outgroup to immigrants is not associated with lower levels of wellbeing, as I initially expected. Furthermore, I have confirmed a strong positive association between social cohesion and higher reported life satisfaction among immigrants. Additionally, in order to exclude the possibility that the null effect might hide a significant interaction, I estimated models that included interaction terms between local ATI and both of these channels. However, the models with interaction terms did not reveal significant variation (not shown). These results do not confirm my hypothesis that the greater variation in ATI across local areas would lead to a stronger association at the most granular level than at the national level (Kogan et al., 2018).

To examine if the association nevertheless varies within England and Wales as hypothesised, I estimated models with regional ATI (Table 4). Model 1 is the unadjusted association, and Model 2 is the full model. Model 3 includes the intergroup friendship measure, and Model 4 includes the interaction term between intergroup friendship and ATI. In contrast to the previous analysis, the association between the regional ATI and immigrants’ life satisfaction is both statistically and substantively significant. The association remains robust even when including additional individual and regional variables. The one-point change in the regional ATI is associated with a 0.181 difference in an individual’s reported life satisfaction (Models 2 and 3). This is twice the difference in reported wellbeing between an employed and unemployed respondent. Considering that the regional ATI vary between 4.7 and 6.2 points, the difference in reported life satisfaction between two individuals with comparable socioeconomic and demographic characteristics can be as high as 0.272 points, depending on their place of residence. This represents a substantial gap.

**Table 4:** Linear regression model estimates of immigrants’ life satisfaction on regional ATI

	Model 1	Model 2	Model 3	Model 4
	Unadjusted	Full model	Channels	Interaction
Regional ATI	0.223**	0.181**	0.180**	0.267**
	(0.087)	(0.092)	(0.092)	(0.111)
Half or less friends same			− 0.054	2.060*
*(r.c. More than half friends same)*			(0.070)	(1.081)
No friends			− 0.580***	− 4.374*
			(0.184)	(2.544)
Half or less friends same × Regional ATI				− 0.372*
*(r.c. More than half friends same)*				(0.190)
No friends × Regional ATI				0.681
				(0.455)
Individual controls		Yes	Yes	Yes
Regional controls		Yes	Yes	Yes
*R*^2^	0.00	0.04	0.05	0.05
*N*	2,096	2,096	2,096	2,096

***p < 0.01; **p < 0.05; *p < 0.1. All analyses are adjusted for sample design and non-response. Controls not shown in the table: sex, age, age squared, education, employment, region of origin, length of stay in the destination, socialisation, and regional unemployment. Full models in Appendix 6

**Fig. 2:**
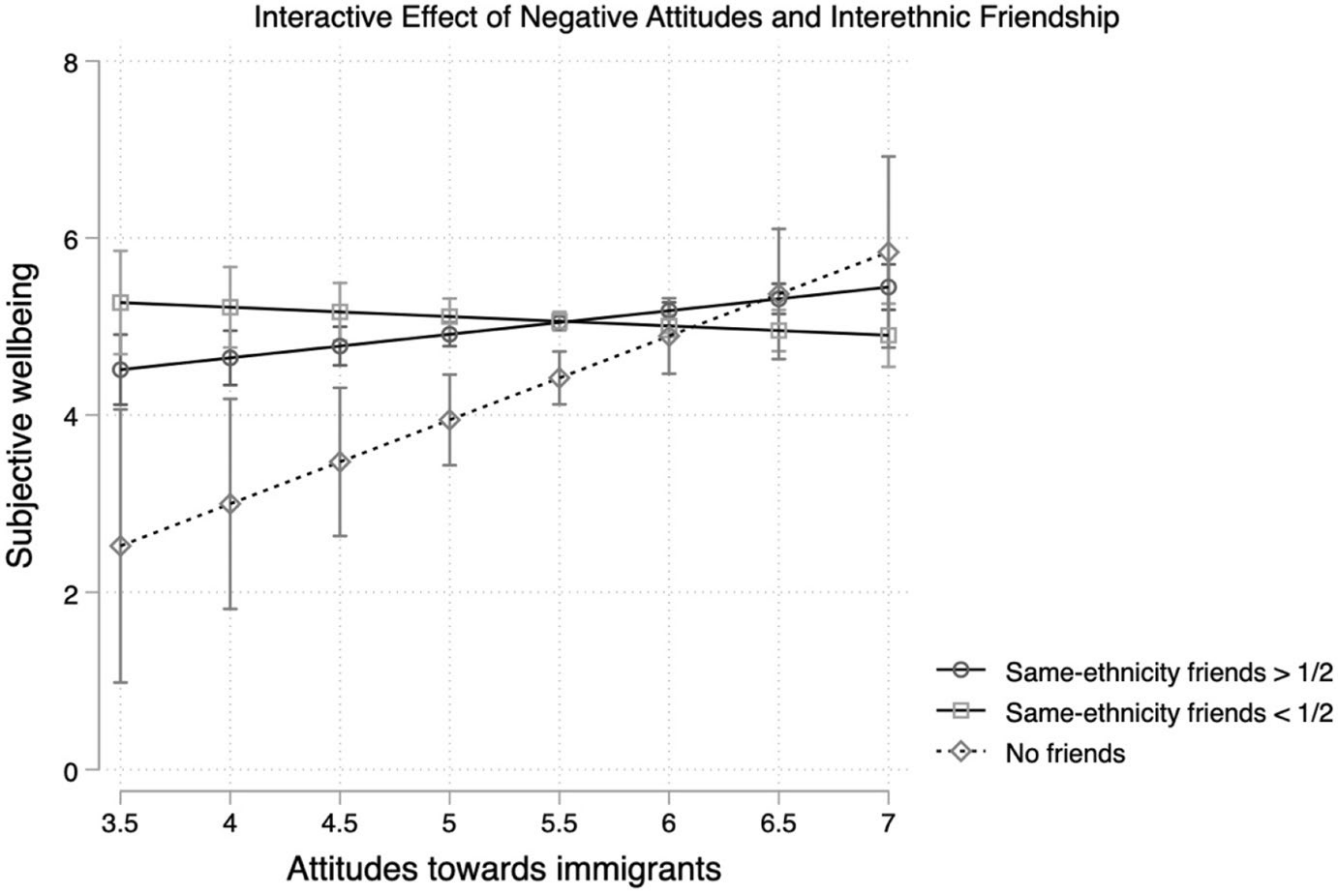
The interacted effect of negative attitudes (GOR level) and respondents' share of interethnic friendships in the destination country. *Note*: Attitudes towards immigrants are measured on a scale from 1 (completely negative) to 10 (completely positive). *Y*-axis shows scale of 3.5–7 for a more detailed view

The inclusion of the channels of exposure in the model does not change the estimated association. Estimates in Model 4 indicate variation in the association between ATI and wellbeing based on interethnic friendship. The main estimates show an association between higher reported wellbeing and a greater share of interethnic friendships. The interaction term shows that friendships have a moderating effect on the association between ATI and wellbeing, meaning that in regions with less positive ATI, the number of interethnic friendships is more important for reporting higher life satisfaction. Estimates show difference of approximately 1 point in reported life satisfaction between those who have half or less friends of the same ethnicity (5.5) and those who have more than half of their friends of the same ethnicity (4.5). Those with no friends report the lowest levels of wellbeing in the regions with the most negative attitudes (2.5). However, despite statistically significance (although *p* < 0.1) and the trend that can be perceived (Fig. 2), the strength of the moderating effect is large only when comparing those with and without friends and is rather low, when focusing on the difference in the respondents’ friends’ ethnicity. The uncertainty is also reflected in the wide confidence intervals. This suggests that interethnic friendships may provide some protection against ATI in regions with more negative observed attitudes, which is in line with my expectations, but does not confirm them fully.

Some of the wellbeing determinants in these models (employment, origin) do not exhibit strong or significant values, despite their widely acknowledged link to wellbeing (Dolan et al., 2008; Paparusso, 2018). Unlike the previous models, I was able to estimate the model for the whole sample, encompassing all NUTS3 regions. My findings indicate that this lack of association is to some extent due to the sample size, as the estimates from the full model show the expected significant associations (not shown).

### Robustness Checks

To assess the robustness of results and the association, I estimated three additional sets of models. First, I ran models using different measures of ATI. Table 5a shows the size and significance of the association between regional ATI and subjective wellbeing estimated through an OLS regression model, defined as Model 2 (Table 4), using two indices of ATI and three ATI measures separately (refer to Table 1). Table 5b shows estimates from the same models run in a logistic regression for all measures except the share of the most negative attitudes. In comparing *average* regional ATI that are reported in the results with the *share, mode*, and *median*, I investigate whether the observed association between regional ATI and wellbeing is driven by individuals with the most negative attitudes. As the results show, there is no link between the share of most negative attitudes and subjective wellbeing. However, considering the strong and significant link with the regional median, I conclude that the overall composition of the attitudes present in a region is more important than the share of the most negative attitudes.

**Table 5:** Comparison of the association between life satisfaction and various measures of regional ATI estimated in the (a) OLS models and (b) logistic regression models

	Welfare	Crime	Jobs	Index 3 ATI measures (10-point scale)	Index 4 ATI measures (5-point scale)
**(a) OLS regression models**					
Average ATI	0.234**	0.136*	0.179**	0.181**	0.359*
	(0.114)	(0.076)	(0.090)	(0.092)	(0.193)
Share of most negative ATI	− 0.008	− 0.009	− 0.015	− 0.010	− 0.009
	(0.007)	(0.008)	(0.012)	(0.009)	(0.009)
Mode ATI	0.039	0.049*	0.040*	–	–
	(0.035)	(0.028)	(0.023)		
Median ATI	0.172**	0.078*	0.069*	0.108**	–
	(0.076)	(0.041)	(0.041)	(0.053)	
*R*^2^	0.05	0.05	0.05	0.05	**0.05**
*N*	2096	2096	2096	2096	**2096**
**(b) logistic regression models**					
Average ATI	0.304**	0.178*	0.230**	0.233**	0.472*
	(0.141)	(0.095)	(0.113)	(0.114)	(0.241)
Mode ATI	0.050	0.062*	0.051*	–	–
	(0.044)	(0.034)	(0.028)		
Median ATI	0.217**	0.102**	0.087*	0.139**	–
	(0.095)	(0.051)	(0.051)	(0.066)	
*N*	2096	2096	2096	2096	**2096**

*p < 0.1; **p < 0.05; ***p < 0.01

Second, I tested my assumption of a linear association between attitudes and wellbeing from the main model, as the association might be limited only to environments with exceptionally positive or negative attitudes. I ran the analyses at the NUTS3 level, dividing the units into three categories based on the degree of negative attitudes. The first model included the first and last quintiles, and the second model included the first and last deciles, representing the most positive and most negative ATI (Appendix 7). These models failed to demonstrate a significant association between ATI and wellbeing. Therefore, I conclude there is no association between local ATI and subjective wellbeing, which is in line with the main results presented in Tables 3, 5a and b.

Lastly, I ran models that included controls for changes in the ethnic composition of the local area over the last 2 years, as these changes might impact local ATI and thus the association. These models also failed to demonstrate a link between immigrants’ wellbeing and local ATI (refer to Appendices 2 and 7). These results further confirm the absence of an association at the local level.

## Discussion

This paper analyses the association between non-migrants’ attitudes towards immigrants and their wellbeing, exploring how this association varies across different aggregated levels of attitudes towards immigrants (ATI), as well as potential channels of exposure. I expected a positive association between welcoming ATI and wellbeing, with a stronger association at a more granular level of aggregation. I also expected that greater social cohesion, ethnic diversity, and more interethnic friendships would have a moderating effect on the negative association. My research introduces an innovative approach by measuring aggregated ATI at multiple spatial levels, aiming to assess whether aggregated ATI are a suitable measure of environmental hostility or hospitality.

Examining this previously unstudied relationship, I demonstrate a strong association between regional ATI and wellbeing, identifying the region as a crucial area for investigating the lived environment of immigrants. Although the majority of immigrants in my sample live in urban areas, and despite the lower variation in ATI across regions compared to local areas, the subjective wellbeing of immigrants is strongly associated with regional differences. However, local ATI did not exhibit a significant association with life satisfaction, and I found no evidence of a link between investigated channels of exposure and the association. My analyses yield three key findings regarding the link between wellbeing and the environment, as measured through ATI.

Firstly, by examining not only the different levels but also the channels of exposure to ATI, I was able to discern whether ATI are specifically linked to immigrants’ personal interactions or if they shape the overall environment in which immigrants live, thereby impacting their life satisfaction beyond their interactions with non-migrants. This is crucial because immigrants might not experience ATI *solely* through contact or exposure. The results at the local level and the absence of moderating effects suggest that ATI are more of a characteristic of the broader environment rather than a function of intergroup contact or exposure. These findings align with theories emphasising the importance of contextual effects and the mean levels of positive/negative intergroup exposure within the environment (Hewstone, 2015). The lack of association at the local level is in line with the person-positivity bias theory (Sears, 1983), which posits that individuals do not channel negative prejudices into their interactions. This also underscores the importance of refocusing research in immigrant studies to encompass *both* the extent and character of intergroup contact or exposure (Esses, 2021) rather than just on one of these elements.

If any importance can be ascribed to immigrants experiencing ATI through channels other than merely contact or exposure, this explains why regional and not local ATI are linked to their wellbeing, as it reflects a broader lived environment. Although these findings do not align with my initial expectation that the association will be stronger at the most granular level, they do confirm that immigrants throughout England and Wales face different levels of hostility from non-migrants, not unlike immigrants residing in different countries (Kogan et al., 2018). The results at the subnational level reveal within-country differences that can be relevant to immigrants’ experience.

Secondly, the analysis of the association using different ATI aggregates reveals that the average value is the most appropriate measure, as it captures the overall composition of local and regional attitudes, which seems to be more relevant for immigrants’ life satisfaction than the share of the most negative ATI. This finding aligns with the contact theory, which posits that individuals with the most negative ATI might not come into contact with immigrants and therefore not expose them to their prejudice. The average value of regional and local ATI does not imply that immigrants are necessarily encountering those on-average-hostile/welcoming non-migrants. However, those values are more reflective of the individual’s experience within their area when compared to national averages.

As I find no evidence of an association with the most negative ATI that is typically linked to perceived discrimination, my findings are also consistent with the hypothesis put forth by Hopkins et al. (2016), which posits that ATI are separate from (perceived) discrimination. Nevertheless, the link between ATI and subjective wellbeing shows that ATI still impact immigrants’ lives. Considering that those with the most negative ATI are also usually voters of right-wing political parties (Malloy et al., 2021), exploring ATI could serve as a complementary approach to investigating perceived discrimination (Safi, 2010; Vohra & Adair, 2000) and voting preferences (Schilling & Stillman, 2021). This could shed light on the cumulative effect of the environment on individuals in their destination country. By employing multiple levels of data aggregation, we can gain insight into the specific levels at which immigrants are exposed to ATI. Moreover, analysing the data at different levels enhances our ability to extrapolate the results to the population to which we can confidently assume we can generalise our results.

Thirdly, the robust association between regional ATI and wellbeing, even after controlling for known predictors of wellbeing, implies a link between the region and wellbeing. This finding is unexpected, as the literature tends to investigate context at the neighbourhood level (Knies et al., 2016; Laurence & Bentley, 2018; Wiedner et al., 2022), which is more comparable to the local area level employed in this paper, or to the policymaking level (the “local turn”) considering potential effects on local residents. Therefore, my research contributes to our understanding of immigrants’ wellbeing by explaining some of the reported variations in life satisfaction observed among different immigrant groups, based on their place of residence.

My results provide evidence that the lived environment is associated with immigrants’ life satisfaction. However, limitations of this study should be acknowledged. First, as this relationship has not yet been studied at the local level, there is a lack of data that would allow establishing the causality and therefore the path of immigrants’ exposure to ATI. The second limitation is that while I have controlled for regions of origin and contextual controls, which accounts for immigrants’ self-selection into regions based on local characteristic and the pull effect of co-ethnics, I cannot completely rule out the potential impact of self-selection on the study results. Immigrants might affect the ATI of non-migrants, for example, causing a more negative ATI towards a particular immigrant group. Lastly, I have controlled only for the potential habituation of individuals to the conditions of the destination country by considering their tenure length. My data do not allow for a definitive determination of whether or not immigrants gradually become accustomed to negative treatment and if this habituation potentially has a protective effect on their wellbeing.

The findings of my study suggest that future research would benefit from examining attitudes on larger samples and by using longitudinal data. It is possible that the lack of association observed in my analysis was driven by lower statistical power due to the sample size, despite only using areas with a pre-defined minimal sample size. Thus, I cannot completely reject the hypothesis that local ATI are associated with (local area determinants of) immigrants’ wellbeing. Longitudinal data would provide valuable tools for conducting such analyses. However, this recommendation is constrained by another limitation related to data availability. First, there is limited data on non-migrant attitudes disaggregated at a small area level in the UK, such as from sources like EVS or the discontinued Citizenship Survey. Second, there is a lack of sufficient datasets that allow for a comprehensive analysis of immigrants. While most immigrants live in urbanised areas, some settle in a much wider variety of other places. By focusing solely on cities in data collection and research, we fail to investigate these other immigrants and create further gaps in understanding the nuances of their experiences. There is great potential for research on ATI and their impact. This cannot be achieved without obtaining more widely available data on ATI and immigrants across countries, not just on those in urban regions.

Nevertheless, my descriptive and exploratory results provide new insights into the relationship between the environment and immigrants’ wellbeing. My study highlights the importance of focusing on variation in the environment within regions and countries. Specifically, I introduce a novel application of the ATI measure as an indicator of the local and regional hostile or welcoming environment, thereby providing a tool for identifying areas where education and integration policies could improve immigrants’ wellbeing by addressing non-migrants’ ATI. The implications of my findings suggest that immigrants residing in different areas of the UK encounter different environments and therefore experience distinct opportunities for wellbeing. Thus, this paper paves the way for future research on the effect of the environment on immigrants.

### Supplementary Information

Below is the link to the electronic supplementary material.

Supplementary file1 (DOCX 63 kb)

## Data Availability

The data that support the findings of this study are available from the UK Data Service (UKDS), but restrictions apply to the availability of these data, which were used under a license for the current study and so are not publicly available. The data are, however, available from the author upon reasonable request and with the permission of UKDS.

## Footnotes

1 While I am aware of the nuanced differences between the terms *subjective wellbeing* and *life satisfaction*, as well as the ongoing discussion regarding these terms, I am using them interchangeably for the purpose of this research.

2 Data are available under restricted access from the UK Data Service and Gesis—Leibniz Institute for the Social Sciences.

3 Individuals in this category are not systematically different from those with valid educational qualifications information.

4 I do not simultaneously control for both ethnicity and area of origin, as those measures are collinear.
