# Supplementary material for: Do Attitudes Towards Immigrants Matter? The Subjective Wellbeing of Immigrants in England and Wales and Their Exposure to Non-migrants
Source: Eur J Popul. 2023 Dec 11;39(1):38. doi: 10.1007/s10680-023-09686-z (PMC10713507; doi:10.1007/s10680-023-09686-z)
Supplement: Supplementary file 1 — Supplementary file1 (DOCX 63 kb) [file 10680_2023_9686_MOESM1_ESM.docx]

**Do attitudes towards immigrants matter? The subjective wellbeing of immigrants in England and Wales and their exposure to non-migrants.**

**Appendices**

1. **Appendix 1 – Variables, missingness and data distribution**

Table A1a

Frequency table for an individual level variable.

| Life satisfaction | Freq. | Percent | Cum. |
| --- | --- | --- | --- |
| Completely unsatisfied | 60 | 2.61 | 2.61 |
| 2 | 131 | 5.71 | 8.32 |
| 3 | 187 | 8.14 | 16.46 |
| 4 | 326 | 14.20 | 30.66 |
| 5 | 461 | 20.08 | 50.74 |
| 6 | 855 | 37.24 | 87.98 |
| Completely satisfied | 276 | 12.02 | 100.00 |
| Total | 2296 | 100.00 |  |
|  | | | |

Table A1b

Frequency table for an individual level variable.

| Sex | Freq. | Percent | Cum. |
| --- | --- | --- | --- |
| Men | 992 | 43.21 | 43.21 |
| Women | 1304 | 56.79 | 100.00 |
| Total | 2296 | 100.00 |  |
|  | | | |

Table A1c

Frequency table for an individual level variable.

| Age | Freq. | Percent | Cum. |
| --- | --- | --- | --- |
| Missing | 3 | 0.13 | 0.13 |
| 17-20 | 35 | 1.52 | 1.66 |
| 21-30 | 238 | 10.37 | 12.02 |
| 31-40 | 525 | 22.87 | 34.89 |
| 41-50 | 560 | 24.39 | 59.28 |
| 51-60 | 411 | 17.90 | 77.18 |
| 61-70 | 313 | 13.63 | 90.81 |
| 71+ | 211 | 9.19 | 100.00 |
| Total | 2296 | 100.00 |  |
| *Note:* This table is only for the purpose of presenting missing data and distribution. Variable age is employed as a continuous variable in the model. | | | |

Table A1d

Frequency table for an individual level variable.

| Place of birth | Freq. | | Percent | Cum. |
| --- | --- | --- | --- | --- |
| Europe, Australia, North America | | 278 | 12.11 | 12.11 |
| India, Pakistan, Bangladesh | | 967 | 42.12 | 54.22 |
| Africa | | 253 | 11.02 | 65.24 |
| South America | | 96 | 4.18 | 69.43 |
| Other | | 702 | 30.57 | 100.00 |
| Total | 2296 | | 100.00 |  |
|  | | | | |

Table A1e

Frequency table for an individual level variable.

| Length of stay in destination | Freq. | Percent | Cum. |
| --- | --- | --- | --- |
| 0-5 years | 96 | 4.18 | 4.18 |
| 6-19 years | 888 | 38.68 | 42.86 |
| 20+ years | 1231 | 53.61 | 96.47 |
| missing | 81 | 3.53 | 100.00 |
| Total | 2296 | 100.00 |  |
|  | | | |

Table A1f

Frequency table for an individual level variable.

| Highest educational qualification | Freq. | Percent | Cum. |
| --- | --- | --- | --- |
| Lower than Lower Secondary | 100 | 4.36 | 4.36 |
| Lower Secondary | 297 | 12.94 | 17.29 |
| Upper Secondary | 181 | 7.88 | 25.17 |
| Higher Education | 531 | 23.13 | 48.30 |
| University | 390 | 16.99 | 65.29 |
| Other | 426 | 18.55 | 83.84 |
| Missing | 371 | 16.16 | 100.00 |
| Total | 2296 | 100.00 |  |
|  | | | |

Table A1g

Frequency table for an individual level variable.

| Worked last week | Freq. | Percent | Cum. |
| --- | --- | --- | --- |
| No | 960 | 41.81 | 41.81 |
| Yes | 1323 | 57.62 | 99.43 |
| Missing | 13 | 0.57 | 100.00 |
| Total | 2296 | 100.00 |  |
|  | | | |

Table A1h

Frequency table for an individual level variable.

| Go out socially | Freq. | Percent | Cum. |  |
| --- | --- | --- | --- | --- |
| No | 402 | 17.51 | 17.51 |  |
| Yes | 1892 | 82.40 | 99.91 |  |
| Missing | 2 | 0.09 | 100.00 |  |
| Total | 2296 | 100.00 |  |  |
|  | | | | |

Table A1i

Frequency table for an individual level variable.

| Proportion of friends of same race | Freq. | Percent | Cum. |
| --- | --- | --- | --- |
| Missing | 5 | 0.35 | 0.35 |
| All | 628 | 27.35 | 27.70 |
| More than half | 810 | 35.28 | 62.98 |
| About half | 431 | 18.77 | 81.75 |
| Less than half | 335 | 14.59 | 96.34 |
| No friends | 84 | 3.66 | 100.00 |
| Total | 2296 | 100.00 |  |
|  | | | |

1. **Appendix 2 – Use of the concurrently collected data**

While consecutive measures – first attitudes towards immigrants and then migrants’ life satisfaction - would be a more fitting approach for approximating the causal impact of attitudes on migrants’ life satisfaction, there are a couple of arguments supporting concurrent measures. First, the empirical research shows that the changes in the attitudes towards immigrants tend to be relatively slow when aggregated (Kaufmann & Harris, 2015), which suggests the average attitudes of non-migrants in the year 2018 can be considered comparable to attitudes in 2017. Second, attitudes tend to be formed during impressionable years, and the significant changes arise between cohorts/generations rather than in the form of short-time individual change (Jeannet & Dražanová, 2019; McLaren & Paterson, 2019).

These two reasons mean that potential difference in attitudes in regions between years is more likely to be driven by the change in the residential composition of areas than by sudden changes in individual attitudes of a significant share of residents. To check the extent of changes in residential composition in the two years, I look at changes in ethnic composition as they are the most relevant for my research. According to the ONS estimates of the ethnic composition in local administrative units of England and Wales, the average absolute change in ethnic composition between 2016 and 2018 was 0.5%. Only 2% of units (8 units) experienced a total change higher than 2%, and the white population share has changed by more than 2% only in 9 units. Considering these changes, I assume changes in other compositions, such as the age structure of residents, are also not sudden. Therefore, knowing that the change in the regions’ compositions in the UK is, first, gradual and, second, that the regional attitudes do not change in the radically opposite direction, it is appropriate to use the concurrently collected data.

1. **Appendix 3 – Correlation and factor analysis for ATI index**

Table A3a

Pairwise correlations of ATI measures aggregated on the NUTS1 level

| Variables | (1) | (2) | (3) | (4) |
| --- | --- | --- | --- | --- |
| (1) Jobs | 1.000 |  |  |  |
|  |  |  |  |  |
| (2) Crime | 0.855 | 1.000 |  |  |
|  | (0.000) |  |  |  |
| (3) Welfare | 0.952 | 0.745 | 1.000 |  |
|  | (0.000) | (0.000) |  |  |
| (4) Development | 0.885 | 0.881 | 0.841 | 1.000 |
|  | (0.000) | (0.000) | (0.000) |  |

(obs=3,118)

Table A3b

Factor analysis/correlation of ATI measures aggregated on the NUTS1 level

Method: principal factors Retained factors = 2
 Rotation: (unrotated) Number of params = 6

| Factor | Eigenvalue | Difference | Proportion | Cumulative |
| --- | --- | --- | --- | --- |
| Factor1 | 3.480 | 3.306 | 0.970 | 0.970 |
| Factor2 | 0.174 | 0.185 | 0.049 | 1.018 |
| Factor3 | -0.011 | 0.044 | -0.003 | 1.015 |
| Factor4 | -0.055 | . | -0.015 | 1.000 |

Factor loadings (pattern matrix) and unique variances

| Variable | Factor1 | Factor2 | Uniqueness |
| --- | --- | --- | --- |
| Jobs | 0.978 | -0.106 | 0.032 |
| Crime | 0.893 | 0.269 | 0.131 |
| Welfare | 0.931 | -0.272 | 0.058 |
| Development | 0.927 | 0.126 | 0.125 |

(obs=3,118)

Table A3c
Pairwise correlations of ATI measures aggregated on the NUTS3 level

| Variables | (1) | (2) | (3) | (4) |
| --- | --- | --- | --- | --- |
| (1) Jobs | 1.000 |  |  |  |
|  |  |  |  |  |
| (2) Crime | 0.772 | 1.000 |  |  |
|  | (0.000) |  |  |  |
| (3) Welfare | 0.752 | 0.827 | 1.000 |  |
|  | (0.000) | (0.000) |  |  |
| (4) Development | 0.591 | 0.783 | 0.733 | 1.000 |
|  | (0.000) | (0.000) | (0.000) |  |

(obs=3,118)

Table A3d

Factor analysis/correlation of ATI measures aggregated on the NUTS level

Method: principal factors Retained factors =2
 Rotation: (unrotated) Number of params = 6

| Factor | Eigenvalue | Difference | Proportion | Cumulative |
| --- | --- | --- | --- | --- |
| Factor1 | 2.944 | 2.890 | 1.046 | 1.046 |
| Factor2 | 0.053 | 0.117 | 0.019 | 1.065 |
| Factor3 | -0.064 | 0.056 | -0.023 | 1.042 |
| Factor4 | -0.119 | . | -0.042 | 1.000 |

Factor loadings (pattern matrix) and unique variances

| Variable | Factor1 | Factor2 | Uniqueness |
| --- | --- | --- | --- |
| Jobs | 0.807 | -0.160 | 0.323 |
| Crime | 0.924 | 0.019 | 0.145 |
| Welfare | 0.889 | -0.023 | 0.209 |
| Development | 0.805 | 0.164 | 0.326 |

(obs=3,118)

Table A3e

Cronbach’s alpha for indices summing 3 and 4 measures aggregated on NUTS1 and NUTS3 level.

| Index | Cronbach’s alpha |
| --- | --- |
| Index of 3 NUTS3 measures | 0.9157 |
| Index of 4 NUTS3 measures | 0.9204 |
| Index of 3 NUTS1 measures | 0.9447 |
| Index of 4 NUTS1 measures | 0.9608 |

1. **Appendix 4 – Ordered logistic regression models**

Table A4

*Ordered logistic regression estimates for models employing NUTS1 index of 3 and 4 aggregated measures of ATI regressed on life satisfaction.*

|  | NUTS1 index of 3 measures | NUTS1 index of 4 measures |
| --- | --- | --- |
| ATI | 0.233 | 0.472 |
|  | (0.114)* | (0.241)+ |
| Gender | 0.103 | 0.103 |
| *(r.c Male)* |  |  |
|  | (0.083) | (0.083) |
| Age | -0.038 | -0.038 |
|  | (0.015)** | (0.015)** |
| Age squared | 0.000 | 0.000 |
|  | (0.000)** | (0.000)** |
| Indian Subcontinent | -0.128 | -0.130 |
| *(r.c.: Global North)* |  |  |
|  | (0.136) | (0.136) |
| Africa | 0.099 | 0.099 |
|  | (0.167) | (0.167) |
| South America | -0.130 | -0.134 |
|  | (0.233) | (0.233) |
| Other | -0.177 | -0.178 |
|  | (0.136) | (0.136) |
| 6-19 years in destination | -0.356 | -0.354 |
| *(r.c.: 0-5 years)* |  |  |
|  | (0.211)+ | (0.211)+ |
| 20+ years in destination | -0.542 | -0.540 |
|  | (0.224)* | (0.224)* |
| Lower secondary education | -0.050 | -0.048 |
| *(r.c. Less than lower secondary)* |  |  |
|  | (0.222) | (0.222) |
| Upper secondary education | -0.251 | -0.247 |
|  | (0.242) | (0.242) |
| Higher education | 0.007 | 0.008 |
|  | (0.211) | (0.211) |
| University | 0.133 | 0.135 |
|  | (0.217) | (0.217) |
| Other | -0.139 | -0.137 |
|  | (0.211) | (0.211) |
| Missing | 0.120 | 0.122 |
|  | (0.216) | (0.216) |
| Employment | 0.049 | 0.048 |
| *(r.c. Employed)* |  |  |
|  | (0.098) | (0.098) |
| Less than ½ friends same ethnicity | -0.048 | -0.047 |
| *(r.c. More than ½ friends same ethnicity)* |  |  |
|  | (0.095) | (0.095) |
| No friends | -0.677 | -0.676 |
|  | (0.229)** | (0.229)** |
| Go out socially | 0.642 | 0.643 |
| *(r.c. No)* |  |  |
|  | (0.111)** | (0.111)** |
| GOR Unemployment rate | 0.067 | 0.069 |
|  | (0.055) | (0.055) |
| Cut 1 | -2.697 | -2.595 |
|  | (0.758)** | (0.812)** |
| Cut 2 | -1.524 | -1.422 |
|  | (0.750)* | (0.805)+ |
| Cut 3 | -0.711 | -0.609 |
|  | (0.748) | (0.804) |
| Cut 4 | 0.132 | 0.233 |
|  | (0.748) | (0.803) |
| Cut 5 | 1.041 | 1.142 |
|  | (0.749) | (0.804) |
| Cut 6 | 3.053 | 3.154 |
|  | (0.751)** | (0.806)** |
| *N* | 2,096 | 2,096 |

1. **Appendix 5 – Full results on the local level**

Table A5

*OLS Regression estimates for models employing NUTS3 ATI measure.*

|  | | Model 1  Unadjusted | | Model 2  Full Model | | Model 3  Full Model with GOR | | Model 4  Channels |  |
| --- | --- | --- | --- | --- | --- | --- | --- | --- | --- |
| Local ATI | | 0.034 | | 0.012 | | 0.015 | | 0.009 |  |
|  | | (0.026) | | (0.027) | | (0.022) | | (0.022) |  |
| Gender | |  | | 0.112 | | 0.111 | | 0.116 |  |
| *(r.c Male)* | |  | |  | |  | |  |  |
|  | |  | | (0.063)* | | (0.062)* | | (0.066)* |  |
| Age | |  | | -0.027 | | -0.030 | | -0.041 |  |
|  | |  | | (0.013)** | | (0.013)** | | (0.012)*** |  |
| Age squared | |  | | 0.000 | | 0.000 | | 0.000 |  |
|  | |  | | (0.000)** | | (0.000)** | | (0.000)*** |  |
| Indian Subcontinent | |  | | -0.090 | | -0.083 | | -0.191 |  |
| *(r.c.: Global North)* | |  | |  | |  | |  |  |
|  | |  | | (0.081) | | (0.083) | | (0.110)* |  |
| Africa | |  | | 0.108 | | 0.080 | | 0.000 |  |
|  | |  | | (0.142) | | (0.145) | | (0.134) |  |
| South America | |  | | -0.215 | | -0.211 | | -0.249 |  |
|  | |  | | (0.191) | | (0.193) | | (0.182) |  |
| Other | |  | | -0.118 | | -0.129 | | -0.128 |  |
|  | |  | | (0.103) | | (0.102) | | (0.110) |  |
| 6-19 years in destination | |  | | -0.182 | | -0.200 | | -0.271 |  |
| *(r.c.: 0-5 years)* | |  | |  | |  | |  |  |
|  | |  | | (0.158) | | (0.164) | | (0.163)* |  |
| 20+ years in destination | |  | | -0.353 | | -0.369 | | -0.440 |  |
|  | |  | | (0.133)** | | (0.141)** | | (0.174)** |  |
| Upper Secondary education | |  | | -0.180 | | -0.200 | | -0.203 |  |
| *(r.c. Lower secondary and less)* | |  | |  | |  | |  |  |
|  | |  | | (0.115) | | (0.117)* | | (0.133) |  |
| Higher education | |  | | -0.013 | | -0.026 | | -0.045 |  |
|  | |  | | (0.108) | | (0.110) | | (0.136) |  |
| University | |  | | 0.081 | | 0.068 | | 0.053 |  |
|  | |  | | (0.089) | | (0.091) | | (0.113) |  |
| Other | |  | | -0.092 | | -0.073 | | -0.103 |  |
|  | |  | | (0.086) | | (0.084) | | (0.116) |  |
| Missing | |  | | 0.042 | | 0.078 | | 0.033 |  |
|  | |  | | (0.117) | | (0.116) | | (0.132) |  |
| Employment | |  | | 0.092 | | 0.095 | | 0.129 |  |
| *(r.c. Employed)* | |  | |  | |  | |  |  |
|  | |  | | (0.058) | | (0.059) | | (0.077)* |  |
| Go out socially | |  | | 0.606 | | 0.598 | | 0.444 |  |
|  | |  | | (0.081)*** | | (0.080)*** | | (0.089)*** |  |
| Share of White British residence | |  | |  | |  | | 0.005 |  |
|  | |  | |  | |  | | (0.003)* |  |
| Social Cohesion | |  | |  | |  | | 0.331 |  |
|  | |  | |  | |  | | (0.043)*** |  |
| Half or less friends same ethnicity | |  | |  | |  | | -0.070 |  |
|  | |  | |  | |  | | (0.070) |  |
| No friends | |  | |  | |  | | -0.440 |  |
|  | |  | |  | |  | | (0.182)** |  |
| *R*^2^ | | 0.00 | | 0.04 | | 0.05 | | 0.08 |  |
| *N* | | 2,096 | | 2,096 | | 2,096 | | 2,096 |  |
|  |  | |  | |  | |  | | |
| * *p*<0.1; ** *p*<0.05; *** *p*<0.01   1. **Appendix 6 – Full results on the regional level**   Table A6  *OLS Regression estimates for models employing NUTS1 ATI measure.*   \|  \| Model 1  Unadjusted \| Model 2  Full Model \| Model 3  Channels \| Model 4  Interaction \| \| --- \| --- \| --- \| --- \| --- \| \| Regional ATI \| 0.223 \| 0.181 \| 0.180 \| 0.267 \| \|  \| (0.087)** \| (0.092)** \| (0.092)** \| (0.111)** \| \| Gender \|  \| 0.112 \| 0.116 \| 0.116 \| \| *(r.c Male)* \|  \|  \|  \|  \| \|  \|  \| (0.067)* \| (0.067)* \| (0.067)* \| \| Age \|  \| -0.029 \| -0.031 \| -0.031 \| \|  \|  \| (0.012)** \| (0.012)*** \| (0.012)*** \| \| Age squared \|  \| 0.000 \| 0.000 \| 0.000 \| \|  \|  \| (0.000)*** \| (0.000)*** \| (0.000)*** \| \| Indian Subcontinent \|  \| -0.098 \| -0.095 \| -0.090 \| \| *(r.c.: Global North)* \|  \|  \|  \|  \| \|  \|  \| (0.110) \| (0.109) \| (0.109) \| \| Africa \|  \| 0.081 \| 0.072 \| 0.067 \| \|  \|  \| (0.136) \| (0.135) \| (0.135) \| \| South America \|  \| -0.225 \| -0.213 \| -0.223 \| \|  \|  \| (0.184) \| (0.183) \| (0.183) \| \| Other \|  \| -0.138 \| -0.136 \| -0.137 \| \|  \|  \| (0.111) \| (0.111) \| (0.111) \| \| 6-19 years in destination \|  \| -0.198 \| -0.200 \| -0.190 \| \| *(r.c.: 0-5 years)* \|  \|  \|  \|  \| \|  \|  \| (0.165) \| (0.165) \| (0.165) \| \| 20+ years in destination \|  \| -0.369 \| -0.376 \| -0.361 \| \|  \|  \| (0.177)** \| (0.176)** \| (0.176)** \| \| Upper secondary education \|  \| -0.191 \| -0.191 \| -0.171 \| \| *(r.c. Lower secondary education)* \|  \|  \|  \|  \| \|  \|  \| (0.135) \| (0.135) \| (0.135) \| \| Higher education \|  \| -0.013 \| -0.022 \| -0.012 \| \|  \|  \| (0.138) \| (0.138) \| (0.138) \| \| University \|  \| 0.074 \| 0.067 \| 0.083 \| \|  \|  \| (0.113) \| (0.114) \| (0.114) \| \| Other \|  \| -0.080 \| -0.076 \| -0.060 \| \|  \|  \| (0.117) \| (0.117) \| (0.117) \| \| Missing \|  \| 0.058 \| 0.040 \| 0.068 \| \|  \|  \| (0.133) \| (0.133) \| (0.134) \| \| Employment \|  \| 0.091 \| 0.093 \| 0.093 \| \| *(r.c. Employed)* \|  \|  \|  \|  \| \|  \|  \| (0.078) \| (0.078) \| (0.078) \| \| Go out socially \|  \| 0.606 \| 0.520 \| 0.523 \| \| *(r.c. No)* \|  \|  \|  \|  \| \|  \|  \| (0.086)*** \| (0.090)*** \| (0.090)*** \| \| GOR Unemployment rate \|  \| 0.037 \| 0.035 \| 0.039 \| \|  \|  \| (0.045) \| (0.045) \| (0.045) \| \| Half or less friends same \|  \|  \| -0.054 \| 2.060 \| \| *(r.c. More than half friends same)* \|  \|  \|  \|  \| \|  \|  \|  \| (0.070) \| (1.081)* \| \| No friends \|  \|  \| -0.580 \| -4.374 \| \|  \|  \|  \| (0.184)*** \| (2.544)* \| \| Half or less friends same x Regional ATI \|  \|  \|  \| -0.372 \| \|  \|  \|  \|  \| (0.190)* \| \| No friends x Regional ATI \|  \|  \|  \| 0.681 \| \|  \|  \|  \|  \| (0.455) \| \| *R*^2^ \| 0.00 \| 0.04 \| 0.05 \| 0.05 \| \| *N* \| 2,096 \| 2,096 \| 2,096 \| 2,096 \| | | | | | | | | | |

* *p*<0.1; ** *p*<0.05; *** *p*<0.01

1. **Appendix 7 – Robustness check models: non-linearity and local ethnic composition change**

Table A8a

*Linear regression estimates for robustness checks models employing NUTS3 ATI measures.*

|  | Model 1  ATI - index | Model 2  ATI - quintiles | Model 3  ATI - deciles | Model 4  Ethnic composition control |
| --- | --- | --- | --- | --- |
| Local ATI | 0.009 |  |  | 0.017 |
|  | (0.022) |  |  | (0.017) |
| 2^nd^-4^th^ quintile |  | -0.014 |  |  |
| *(r.c. 1^st^ quintile)* |  |  |  |  |
|  |  | (0.091) |  |  |
| 5^th^ quintile |  | 0.106 |  |  |
|  |  | (0.082) |  |  |
| 2^nd^-9^th^ decile |  |  | -0.122 |  |
| *(r.c. 1^st^ decile)* |  |  |  |  |
|  |  |  | (0.061)+ |  |
| 10^th^ decile |  |  | 0.038 |  |
|  |  |  | (0.073) |  |
| Change in 2 years |  |  |  | 0.051 |
|  |  |  |  | (0.058) |
| Gender | 0.116 | 0.115 | 0.114 | 0.117 |
| *(r.c Male)* |  |  |  |  |
|  | (0.066)+ | (0.065)+ | (0.065)+ | (0.066)+ |
| Age | -0.041 | -0.041 | -0.041 | -0.041 |
|  | (0.012)** | (0.012)** | (0.012)** | (0.012)** |
| Age squared | 0.000 | 0.000 | 0.000 | 0.000 |
|  | (0.000)** | (0.000)** | (0.000)** | (0.000)** |
| Indian Subcontinent | -0.192 | -0.182 | -0.191 | -0.190 |
| *(r.c.: Global North)* |  |  |  |  |
|  | (0.086)* | (0.086)* | (0.087)* | (0.086)* |
| Africa | -0.001 | 0.005 | 0.008 | 0.003 |
|  | (0.140) | (0.142) | (0.141) | (0.141) |
| South America | -0.249 | -0.243 | -0.243 | -0.247 |
|  | (0.185) | (0.184) | (0.186) | (0.186) |
| Other | -0.127 | -0.122 | -0.122 | -0.127 |
|  | (0.098) | (0.098) | (0.098) | (0.098) |
| 6-19 years in destination | -0.269 | -0.267 | -0.279 | -0.272 |
| *(r.c.: 0-5 years)* |  |  |  |  |
|  | (0.158) | (0.159) | (0.159)+ | (0.159)+ |
| 20+ years in destination | -0.438 | -0.439 | -0.455 | -0.442 |
|  | (0.129)** | (0.129)** | (0.130)** | (0.130)** |
| Upper Secondary education | -0.201 | -0.196 | -0.195 | -0.197 |
| *(r.c. Lower secondary and less)* |  |  |  |  |
|  | (0.117)+ | (0.116) | (0.117) | (0.118) |
| Higher education | -0.045 | -0.041 | -0.035 | -0.042 |
|  | (0.108) | (0.108) | (0.111) | (0.109) |
| University | 0.055 | 0.056 | 0.061 | 0.059 |
|  | (0.089) | (0.089) | (0.090) | (0.091) |
| Other | -0.101 | -0.100 | -0.105 | -0.101 |
|  | (0.084) | (0.083) | (0.084) | (0.084) |
| Missing | 0.033 | 0.034 | 0.040 | 0.037 |
|  | (0.120) | (0.120) | (0.121) | (0.120) |
| Employment | 0.128 | 0.126 | 0.126 | 0.130 |
| *(r.c. Employed)* |  |  |  |  |
|  | (0.061)* | (0.061)* | (0.060)* | (0.062)* |
| Go out socially | 0.443 | 0.440 | 0.440 | 0.445 |
|  | (0.089)** | (0.090)** | (0.090)** | (0.089)** |
| Share of White British residence | 0.005 | 0.005 | 0.005 | 0.005 |
|  | (0.002)** | (0.002)** | (0.002)** | (0.002)** |
| Social Cohesion | 0.331 | 0.331 | 0.332 | 0.329 |
|  | (0.041)** | (0.041)** | (0.042)** | (0.041)** |
| All same friends | 0.001 | 0.002 | 0.003 | 0.003 |
|  | (0.066) | (0.065) | (0.065) | (0.066) |
| More than half same | -0.050 | -0.045 | -0.043 | -0.048 |
|  | (0.090) | (0.091) | (0.090) | (0.091) |
| About half the same | -0.097 | -0.093 | -0.089 | -0.095 |
|  | (0.097) | (0.098) | (0.099) | (0.098) |
| Less than half same | -0.440 | -0.444 | -0.446 | -0.441 |
|  | (0.218)+ | (0.219)+ | (0.219)+ | (0.218)+ |
| *R*^2^ | 0.08 | 0.08 | 0.08 | 0.08 |
| *N* | 2,096 | 2,096 | 2,096 | 2,096 |

+ *p*<0.1; * *p*<0.05; ** *p*<0.01, these models control for the GOR (dummy), which is not shown in the table
